# A Cluster Randomized Trial of Routine HIV-1 Viral Load Monitoring in Zambia: Study Design, Implementation, and Baseline Cohort Characteristics

**DOI:** 10.1371/journal.pone.0009680

**Published:** 2010-03-12

**Authors:** John R. Koethe, Andrew O. Westfall, Dora K. Luhanga, Gina M. Clark, Jason D. Goldman, Priscilla L. Mulenga, Ronald A. Cantrell, Benjamin H. Chi, Isaac Zulu, Michael S. Saag, Jeffrey S. A. Stringer

**Affiliations:** 1 Centre for Infectious Disease Research in Zambia, Lusaka, Zambia; 2 Division of Infectious Diseases, Vanderbilt University School of Medicine, Nashville, Tennessee, United States of America; 3 Department of Obstetrics and Gynecology, University of Alabama at Birmingham School of Medicine, Birmingham, Alabama, United States of America; 4 Department of Internal Medicine, University Teaching Hospital, Lusaka, Zambia; 5 Division of Infectious Diseases, University of Alabama at Birmingham School of Medicine, Birmingham, Alabama, United States of America; Instituto de Pesquisa Clinica Evandro Chagas, FIOCRUZ, Brazil

## Abstract

**Background:**

The benefit of routine HIV-1 viral load (VL) monitoring of patients on antiretroviral therapy (ART) in resource-constrained settings is uncertain because of the high costs associated with the test and the limited treatment options. We designed a cluster randomized controlled trial to compare the use of routine VL testing at ART-initiation and at 3, 6, 12, and 18 months, versus our local standard of care (which uses immunological and clinical criteria to diagnose treatment failure, with discretionary VL testing when the two do not agree).

**Methodology:**

Dedicated study personnel were integrated into public-sector ART clinics. We collected participant information in a dedicated research database. Twelve ART clinics in Lusaka, Zambia constituted the units of randomization. Study clinics were stratified into pairs according to matching criteria (historical mortality rate, size, and duration of operation) to limit the effect of clustering, and independently randomized to the intervention and control arms. The study was powered to detect a 36% reduction in mortality at 18 months.

**Principal Findings:**

From December 2006 to May 2008, we completed enrollment of 1973 participants. Measured baseline characteristics did not differ significantly between the study arms. Enrollment was staggered by clinic pair and truncated at two matched sites.

**Conclusions:**

A large clinical trial of routing VL monitoring was successfully implemented in a dynamic and rapidly growing national ART program. Close collaboration with local health authorities and adequate reserve staff were critical to success. Randomized controlled trials such as this will likely prove valuable in determining long-term outcomes in resource-constrained settings.

**Trial Registration:**

Clinicaltrials.gov NCT00929604

## Introduction

The rapid expansion of access to antiretroviral therapy (ART) in sub-Saharan Africa has led to dramatic drops in AIDS-related mortality in a variety of settings, [1], [2], [3], [4], [5] but a tremendous unmet need for HIV care remains. [6] Limited healthcare infrastructure, personnel, and funding create a tension between the twin goals of expanding access to ART and optimizing care for those already receiving treatment. Arguments to minimize sophisticated laboratory monitoring in favor of treatment program expansion [7] must be weighed against the potential for improved outcomes and cost savings associated with better tools for monitoring treatment. [8]

The measurement of HIV-1 RNA levels (*i.e.,* viral load [VL]) is recommended to monitor the response to ART in developed countries. [9], [10] The World Health Organization (WHO) does not recommend routine VL testing in resource-constrained settings, in part due to the cost and complex infrastructure needed for reliable results. [11] In these settings, WHO has proposed the use of clinical and CD4^+^ lymphocyte-based criteria to guide treatment decisions. However, multiple studies have demonstrated the poor performance of these criteria in sub-Saharan Africa and the frequent discordance between immunologic and virologic responses to ART. [12], [13], [14], [15]

Given the lack of third-line ART regimens in much of sub-Saharan Africa and the high cost, sophisticated laboratory equipment, and technical training necessary to perform VL testing, the widespread adoption of this technology must be informed by solid evidence. To obtain these data, we implemented a large clinical trial investigating the public health impact of routine virologic monitoring on patient outcomes in Lusaka, Zambia. A clinic-level, cluster-randomized design was selected as most appropriate from a logistical and ethical perspective. In this report, we describe the study design, statistical considerations, baseline characteristics of the cohort, and our experience in implementing a large clinical trial in a resource-constrained setting.

## Methods

The protocol for this trial and supporting CONSORT checklist are available as supporting information; see Checklist S1 and Protocol S1.

### Ethics Statement

This study was conducted according to the principles expressed in the Declaration of Helsinki. The study protocol and consent documents were approved by the University of Zambia Research Ethics Committee (reference number 002-04-06) and the University of Alabama at Birmingham institutional review board (reference number X060707001). Written informed consent was obtained from all adult participants; no minors were enrolled in the study.

### Study Design

**Figure 1:**
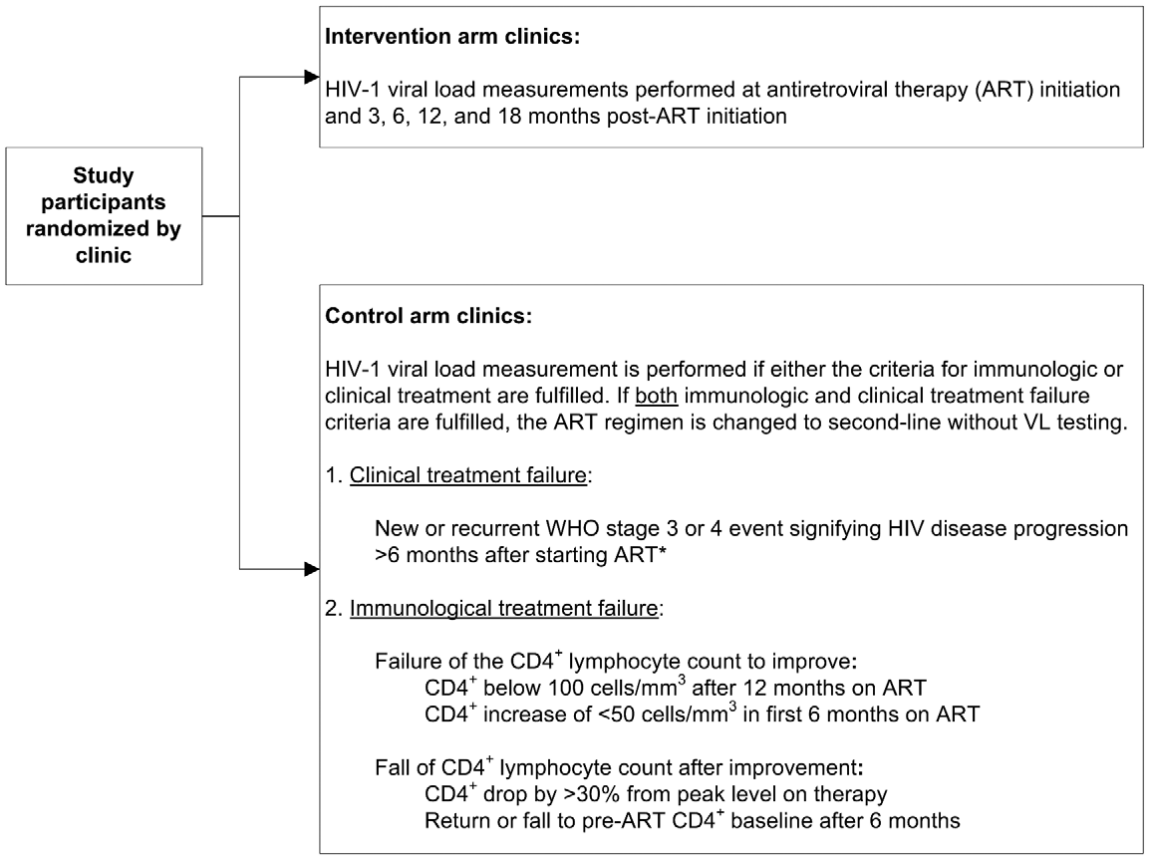
Design of the Viral Load Study. * Immune Reconstitution Syndrome (IRIS) is not considered evidence of clinical treatment failure. The assessment of whether a clinical event represents IRIS or a genuine incident opportunistic infection is determined locally by the clinician. Note: when clinical or immunologic criteria for therapeutic failure are met, a procedure for allocating ‘discretionary’ viral load testing is utilized. Any evident infections are investigated and treated. In cases of suspected immunologic treatment failure, the CD4^+^ lymphocyte count is repeated one month after treatment of infection and/or intensive adherence counseling. If the patient still meets criteria for therapeutic failure after adherence is judged to be excellent and (in cases of immunologic failure) after a repeat CD4^+^ lymphocyte count, HIV-1 viral load testing is performed for those patients meeting either clinical or immunologic criteria, but not both. Patients meeting both clinical and immunologic criteria for therapeutic failure are assumed to have virologic failure and the decision to change to ART regimen is made without viral load testing.

‘Effectiveness of HIV Viral Load Monitoring on Patient Outcome in Resource-Poor Settings’ - known locally as the Viral Load Study or VLS - is a two-arm, clinic-level cluster randomized trial to evaluate the use of routine plasma HIV-1 VL monitoring to improve survival and decrease HIV disease progression in patients initiating ART in Lusaka, Zambia. Participants enrolled in the study intervention arm of VLS receive VL testing at ART initiation and at 3, 6, 12, and 18 months post-initiation, and the results are provided to the clinician for the purpose of patient care. Participants in the study control arm receive ‘discretionary’ viral load testing according to local guidelines: VL testing is performed for those patients meeting either clinical or immunologic criteria for treatment failure, but not both. Patients meeting both clinical and immunologic criteria for therapeutic failure are assumed to have virologic failure, and VL testing is not performed. Discretionary viral load testing was performed on fewer than 7% of all patients in the ART program in 2008. Participants in the control arm have blood drawn according to the same schedule as the intervention arm, but the samples are frozen and archived. Aside from routine VL testing (in the intervention arm) or phlebotomy (in the control arm), VLS participants receive local standard medical care. Figure 1 describes the study design.

The primary aim of VLS is to compare mortality at 18 months among ART-naïve patients initiating ART and receiving care at facilities with access to routine VL testing, compared to those initiating first regimens and receiving the current standard of care (*i.e.,* discretionary VL testing). The secondary objectives are (1) to compare select indicators of clinical disease progression in the two comparison groups (*e.g.,* CD4+ lymphocyte response, incident opportunistic infections, and weight loss); (2) to assess the impact of more rapid ART regimen switching on available second and third-line treatment options; (3) to monitor the effectiveness of newer antiretroviral medications (principally TDF/FTC); (4) to characterize the timing and sequence of HIV drug resistance development among patients in each arm; and (5) to assess the feasibility, acceptability, and cost effectiveness of the two management strategies in a resource-constrained sub-Saharan African setting.

A cluster-randomized design was selected as most appropriate from a logistical perspective to facilitate VL sample collection and reporting of results, and from an ethical perspective to minimize the perception among control arm participants that he/she was receiving ‘less’ treatment. The VLS was designed as a “pragmatic” trial in which the intervention to be tested is overlaid on the background of usual clinical practice, with a minimum of study-related practice constraints, liberal inclusion, and few exclusion criteria.

### Location and Personnel

The severity and challenges of the HIV epidemic in Zambia are typical of many sub-Saharan Africa countries. Fifteen percent of adults (15–49 years old) are estimated to be HIV-infected [16] and 64% of the population of 11.7 million live on less than 1.25 US dollars per day. [17] The Zambian national program for HIV care and treatment was implemented in Lusaka's public health sector in April 2004 and has expanded rapidly across the country. By May 2009, 198,000 patients were enrolled in HIV care at 67 sites, and 127,000 had started ART. Clinical care in the Zambian national ART program has been previously described. [1], [18] Briefly, HIV-infected patients undergo a history and physical examination, WHO disease staging, and a CD4^+^ lymphocyte count at enrollment. Patients with WHO stage 4 disease; a CD4^+^ lymphocyte count <200 cells/mm^3^; or WHO stage 3 disease and a CD4^+^ lymphocyte count <350 mm^3^ are eligible to initiate ART. We have previously described the Zambian national guidelines for determining clinical and immunologic treatment failure (see also figure 1). [19] Plasma HIV-1 RNA measurements, where available, are used sparingly to adjudicate uncertain presentations.

When VLS began enrollment in December 2006, the first-line ART regimen was a non-nucleoside reverse transcriptase inhibitor (NNRTI), either efavirenz (EFV) or nevirapine (NVP), in combination with two nucleoside reverse transcriptase inhibitors (NRTIs): lamivudine (3TC) with either zidovudine (ZDV) or stavudine (d4T). In July 2007, the NRTI combination of tenofovir (TDF) and emtricitabine (FTC) or 3TC was introduced as first line therapy. Patients on treatment prior to July 2007 remained on the original regimen, except in cases of treatment failure or toxicity.

VLS employed dedicated study staff at each clinical site to ensure the proper follow-up of participants without detracting from routine patient care. A clinic-based study nurse at each site saw participants at every visit and collected serum specimens for VL testing and archiving within a 60-day window around each sampling point. An additional pool of four nurses provided support or replacement staffing as needed. Each study facility also employed a clinical assistant, who coordinated patient movement through the clinic and ensured that study participants saw the study nurse. Additionally, the clinical assistant worked with clinic-based community volunteers to ensure good participant follow-up.

### Recruitment and Screening

We recruited participants from 12 Lusaka district clinics. The rationale for selection of study clinics is described below in *statistical considerations*. The control and intervention arms were each allocated six clinics. Adult patients with a documented HIV-1 infection presenting for medical care at participating clinics were referred for screening by the VLS nurse based at each site. Study nurses and participants were not blinded to the allocation of the study clinic at the time of screening.

Patients were eligible to enroll in the study if they qualified for ART per Zambian national guidelines and were initiating treatment on the day of study enrollment; resided in the geographical catchment area of the VLS clinic and intended to remain in the area for the duration of study; agreed to adhere to the study visit schedule and to be followed-up at home in the event of a missed study visit; and provided informed consent and a baseline blood draw. Patients were ineligible if they reported receipt of more than 7 days (cumulative) of prior ART in the past, with the exception of ZDV prophylaxis or single dose NVP for prevention of mother-to-child transmission (PMTCT). We also excluded those with any exposure to ART in the prior month, those with any condition that in the opinion of the study staff would interfere with adherence to study requirements (*e.g.,* mental illness or active drug use or alcohol dependence), those who required hospital referral for a serious illness at the time of treatment initiation; and those who refused or were unable to provide consent to participate.

Many roads in Lusaka residential neighborhoods are unmarked, and each participant was requested to provide a mobile phone number (if available), and instructions or a diagram for locating their primary residence. Contact information for a close associate was also requested. The study nurse updates the locator form at each visit, and clinical associates use this information to trace participants lost to follow-up.

### Data Collection

Study data are collected in the national programmatic database and a dedicated research database. Following every patient visit, data associates enter a range of information into the *SmartCare* electronic medical record system (http://www.smartcare.org.zm) developed by the Zambian Ministry of Health, the Centers for Disease Control and Prevention, and the Centre for Infectious Disease Research in Zambia. VLS participant information is also entered into the separate research database. A computer program routinely compares the programmatic and research databases, and all discrepancies are reconciled in the research database after reference to the paper chart.

VLS participant serum specimens are separated at the facility level from programmatic specimens and transported to a central laboratory by daily courier. HIV-1 VL is measured by the Roche Amplicor HIV-1 RNA Monitor kit (version 1.5; Roche Molecular Diagnostics, Pleasanton, CA, USA). CD4^+^ lymphocyte counts are performed using a Beckman Coulter flow cytometer (Beckman Coulter, Inc., Fullerton, CA, USA); chemistry assays using an Olympus AU400 (Olympus Diagnostics, Hamburg, Germany); and hemogram or complete blood count with differential using a Horiba ABX Pentra 80 (Horiba ABX Diagnostics Inc., Montpellier, France). The study maintains a specimen archive containing whole blood from ART-initiation and plasma from each subsequent collection point for all participants.

### Statistical Considerations

Twelve ART clinics in Lusaka, Zambia constituted the units of randomization. In order to maximize the comparability of patients across clinics and to limit the effect of clustering within clinics (*i.e.,* to lower the intraclass correlation coefficient), we stratified clinics into pairs according to matching criteria. One clinic in each pair was selected by a computerized randomization program to implement VL monitoring as the standard of care (table 1). The stratification of clinics was based primarily on estimated 18-month mortality rates, but we also considered the duration of operation and the number of active patients (*i.e.,* patients with a clinic or pharmacy visit in the preceding 90 days).

**Table 1:** Matched study clinic pairs (As of December 1, 2006, prior to study commencement).

Study clinic	Study arm	Duration of operation (months)	Number of adult patients on ART*	Probability of survival at 18 months (95%CI)†
Matero Ref.	Intervention	28.0	2,375	0.86 (0.85, 0.88)
Kanyama	Control	31.1	2,728	0.88 (0.86, 0.89)
Chipata	Intervention	21.9	1,151	0.82 (0.80, 0.85)
Chawama	Control	9.0	775	0.92 (0.90, 0.95)
George	Intervention	28.0	1,460	0.87 (0.85, 0.89)
Chilenje	Control	27.0	1,097	0.85 (0.83, 0.87)
Mtendere	Intervention	31.1	1,044	0.85 (0.83, 0.87)
Bauleni	Control	23.4	456	0.82 (0.78, 0.86)
Kabwata	Intervention	8.5	297	0.95 (0.90, 0.99)
Matero Main	Control	4.0	227	0.91 (0.85, 0.96)
Ngombe‡	Intervention	0	0	na
Makeni‡	Control	0	0	na

*Excludes former patients classified as deceased or lost to follow-up.

† Kaplan-Meier estimate of the proportion of adult patients surviving at 18 months post-ART initiation.

‡ Ngombe and Makeni clinics opened in March 2007.

Six study clinics (Bauleni, Chawama, Chilenje, Chipata, George, and Mtendere) were operating for over 18 months at the time of randomization (range: 20 to 30 months as of October 1, 2006), and provided an estimated average 18-month mortality rate of 15.6 per 100 person-years. This approximates the overall mortality rate of 16.1 per 100 person-years previously observed in the national ART program. [1] However, the mortality rate varied from 13.1 per 100 person-years at George to 20.4 per 100 person-years at Chipata, illustrating the need to account for dependence between subjects within clinics. The calculated coefficient of variation, *k*, for mortality rates at these 6 clinics was 0.14.

The six additional study clinics were not included in the calculation of the coefficient of variation for 18-month mortality. Matero Reference and Kanyama clinics were selected for inclusion in the study after the average 18-month mortality was calculated, and the pairing was based on a similar duration of operation (26 and 30 months, respectively) and number of active patients (3,492 and 4,314, respectively). Matero Main and Kabwata clinics were matched based on a similarly short duration of operation (2 and 7 months, respectively) and smaller active patient population (357 and 525, respectively). Lastly, Makeni and Ngombe clinics were matched as both clinics were scheduled to open after the study commenced in December 2006.

**Figure 2:**
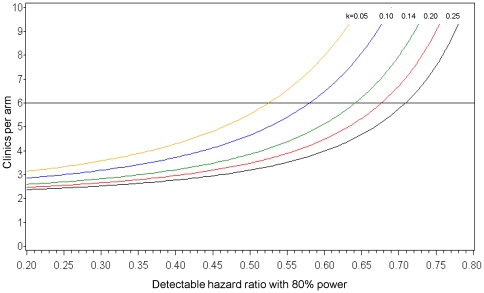
Detectable hazard ratio as a consequence of utilizing routine HIV-1 viral load monitoring at varying between-clinic coefficients of variation. Calculation assumes a historical 18 month post-ART mortality rate of 15.6 per 100 years (140 patients remaining per clinic).

The study was powered to detect a hazard ratio of 0.64 or lower as a consequence of utilizing routine VL monitoring in clinical care, which represented a 36% reduction in mortality (15.6 per 100 person-years versus 10.0 per 100 person years). Matching clinics will permit the use of a matched *k* in future analyses, which should be lower than the unmatched *k* and may improve study power. Using our estimated unmatched *k* (0.14) as a conservative estimate of the matched *k*, 1680 participants (140 per clinic; alive or deceased) will need to remain in the study after 18 months of follow-up to maintain the sample size assumed in the power calculations. [20] Figure 2 shows the detectable hazard ratio (alpha of 0.05 and beta of 0.20) at different coefficients of variation.

The primary analysis will compare mortality at 18 months between the intervention and control arms, while taking into account the design-based cluster matching and the testing of the intervention effect over all community pairs. The difference in mortality for each matched pair will be computed and statistical significance will be assessed using a paired t-test or the non-parametric rank sum test. While matching on clinic characteristics is expected to produce balance with respect to clinic related factors, imbalances in other covariates (*e.g.,* socio-economic status, age distribution) will be adjusted at the cluster level using an extension of the Mantel-Haenzsel test.

We calculated an enrollment target of 2100, or 175 participants per clinic, by assuming an attrition rate (voluntary withdrawals and loss to follow-up) of 20% at 18 months based on historical estimates. We expected to have at least 1680 participants (alive or deceased) remaining in the study cohort at completion.

Patients who move from one study clinic catchment area to another study clinic area are continued in their original assigned study arm, regardless of the assignment of the second clinic. Patients who move to areas served by non-study clinics are encouraged to continue attending the study clinic, but are classified as withdrawn if they do not.

Brief treatment interruptions are not uncommon among ART patients in Lusaka, often due to familial or economic factors. Patients in the national ART program, and VLS participants, are classified as lost to follow-up when (1) >37 days late for a scheduled pharmacy visit, or (2) do not to return within 60 days of the last clinical visit if no pharmacy visit was scheduled. However, participants classified as lost to follow-up have the option to re-enter the study if they return to care within 120 days.

## Results

**Figure 3:**
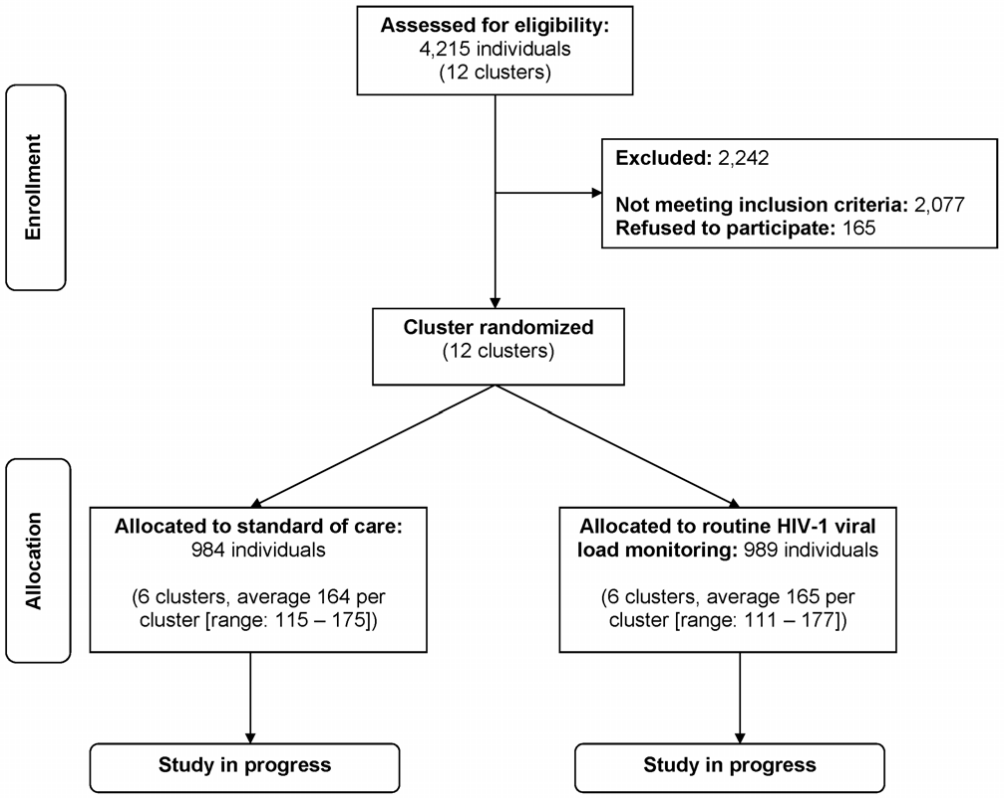
Participant screening and enrollment.

**Figure 4:**
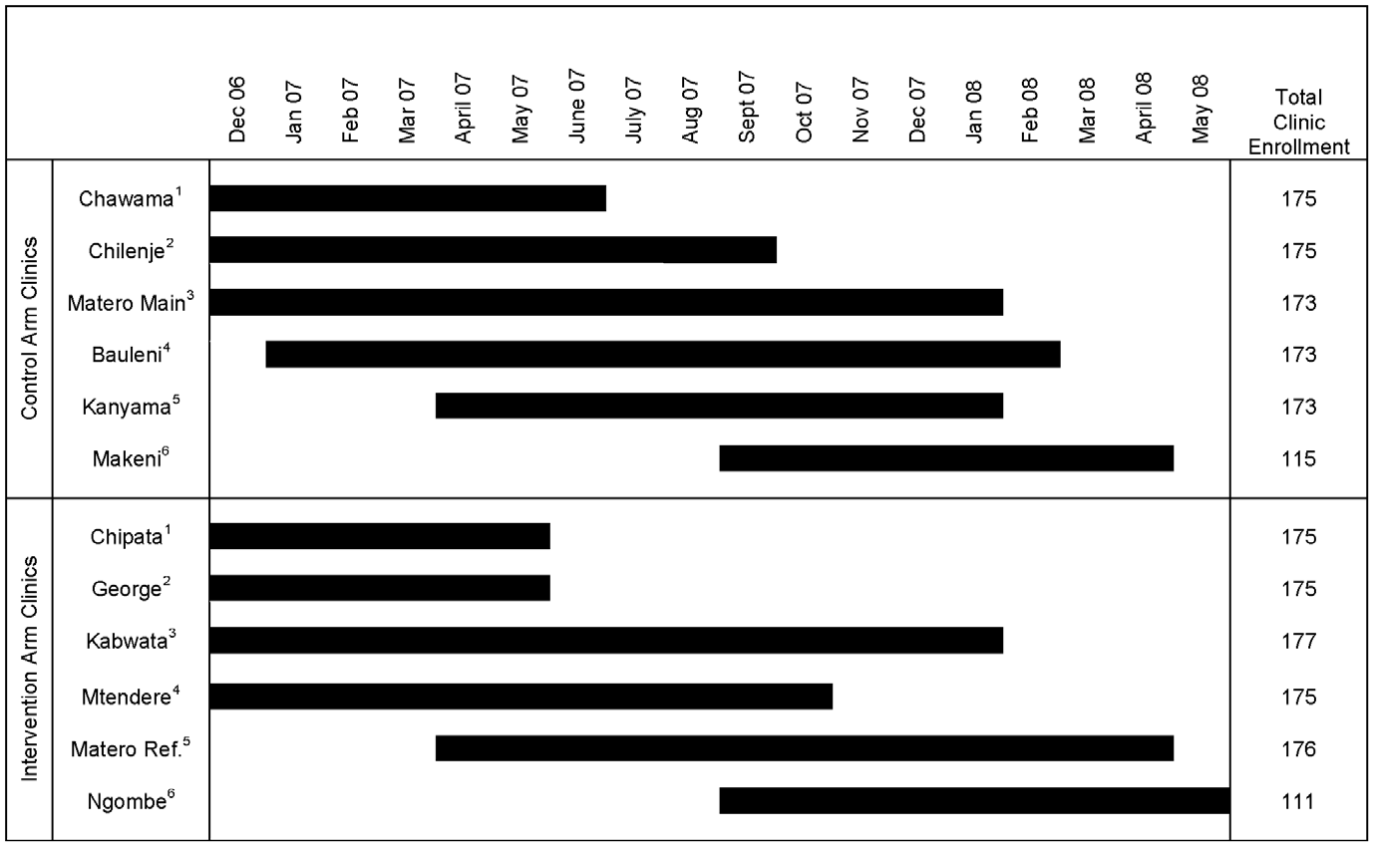
Participant accrual by clinic. Superscript denotes matched clinic pairs. ‘Matero Ref.’ refers to Matero Reference clinic.

Between December 2006 and May 2008, we enrolled 1973 participants at 12 clinics (figure 3). This represents 47% of the 4,215 patients screened and 26% of the 7,723 patients initiating ART at participating study sites. Enrollment was staggered by clinic pairs to permit close staff support (Figure 4). Ten of the 12 sites were fully enrolled. Enrollment at Ngombe and Makeni was halted after 111 and 115 participants, respectively, owing to lower than anticipated patient volumes at each site.

Demographic characteristics of participants in each arm are shown in Table 2. Overall, the mean age was 34.6, 61% were female, 78% had a CD4^+^ lymphocyte count <200 cells/mm^3^, 14% had a CD4^+^ lymphocyte count <50 cell/mm^3^, and 68% had WHO stage 3 or 4 disease at enrollment. The first-line ART regiments included NVP (74%) or EFV (25%), and ZDV (40%), d4t (36%) or TDF (24%). There were no significant differences in measured baseline characteristics between study arms (p<0.05).

**Table 2:** Baseline characteristics of participants by study arm.

	Control Arm (N = 984)		Intervention Arm (N = 989)	
	N	Value	N	Value
Age, mean years (sd)	984	34.3 (8.4)	989	34.8 (8.3)
Sex				
Female	600	61.0%	584	59.0%
Male	384	39.0%	405	41.0%
Viral Load, mean log_10_ copies/mL (sd)			988	5.1 (0.8)
≤100,000 copies/mL	NA		358	36.2%
>100,000 copies/mL	NA		630	63.8%
Adherence Support				
No	13	1.3%	25	2.5%
Yes	971	98.7%	964	97.5%
CD4^+^ Lymphocyte Count, mean cells/mm^3^ (sd)	957	146 (82.6)	941	145 (86.8)
≥200 cells/mm^3^	194	20.3%	223	23.7%
50–199 cells/mm^3^	652	68.1%	572	60.8%
<50 cells/mm^3^	111	11.6%	146	15.5%
WHO Stage				
I or II	308	32.8%	295	31.1%
III	561	59.7%	560	59.1%
IV	70	7.5%	93	9.8%
Hemoglobin, mean g/dL (sd)	952	10.9 (2.1)	944	11.0 (2.1)
≥8.0 g/dL	878	92.2%	879	93.1%
<8.0 g/dL	74	7.8%	65	6.9%
Body Mass Index, mean kg/m^2^ (sd)	962	20.4 (3.4)	960	20.3 (3.7)
≥16 kg/m^2^	894	92.9%	884	92.1%
<16 kg/m^2^	68	7.1%	76	7.9%
Creatinine Clearance, mean mL/min (sd)*	894	59.7 (26.4)	929	59.7 (22.4)
Normal	703	78.8%	713	76.9%
Abnormal	189	21.2%	214	23.1%
Alanine Aminotransferase, mean u/L (sd)**	902	22.4 (18.0)	936	23.1 (16.2)
Normal	869	96.3%	899	96.0%
Abnormal	33	3.7%	37	4.0%
Anti-Tuberculosis Therapy				
No	807	82.0%	833	84.2%
Yes	177	18.0%	156	15.8%
Antiretroviral Regimen				
ZDV + 3TC + NVP	345	35.2%	359	36.3%
ZDV + 3TC + EFV	44	4.5%	46	4.7%
D4T + 3TC + NVP	275	28.1%	314	31.8%
D4T + 3TC + EFV	65	6.6%	52	5.3%
TDF + FTC + NVP	114	11.6%	57	5.8%
TDF + FTC + EFV	134	13.7%	157	15.9%
Other	2	0.2%	3	0.3%

Note: missing baseline values and values not collected within the required time period are not shown.

*Normal creatinine clearance (calculated using the Cockcroft-Gault equation) ≥90 mL/min.

**Normal alanine aminotransferase <62.5 u/L.

## Discussion

The implementation of a large clinical trial of HIV-1 VL monitoring in the context of a dynamic, rapidly growing national ART program is challenging but feasible. Our experience can inform investigators working in similar settings. The VLS will provide a range of critical data on the role of virologic monitoring of patients on ART in resource-constrained settings, and the specimen archive offers a unique opportunity to investigate a host of future research questions. Randomized controlled trials such as the VLS will likely prove valuable in determining long-term outcomes in resource-constrained settings.

This study represents a successful collaboration between the study team and the national ART program during a period of rapid expansion. Investigators and trial administrators need the resources and reserve capacity to adjust staffing levels in response to deficiencies, and must avoid diverting personnel from the national program while also delineating study staff responsibilities in an environment of competing needs. In selecting study staff, candidates with experience in government ART clinics are preferred, but senior management must closely monitor all staff initially to address deficiencies and ensure the collection of study data and specimens within the specified time period. Close communication between investigators and district health managers, and attention to disagreements between study staff and ART clinic staff, are essential to promote prompt resolution and improve study integration. Early provision of informational materials and/or presentations to non-study clinic staff may increase the general perception that the study is relevant and an appropriate use of resources.

Our clinic-level, cluster randomization design accounted for the lack of independence of subjects within clinic populations. Matching clinics according to facility-level characteristics yielded a study cohort without significant differences in baseline participant characteristics in each arm. However, our method may have missed important differences in the patient populations served by each clinic (*e.g.,* socio-economic status), and insufficient randomization may result in the emergence of confounding variables as the study progresses. Additionally, the baseline characteristics of participants remaining in each arm at study conclusion will need to be compared to determine potential selection effects of the intervention versus study participation alone. A coefficient of variation for overall 18-month mortality rate among study clinics, and a matched *k*, will be calculated at study conclusion to determine the final detectable hazard ratio.

Under-enrollment in one clinic-pair (Ngombe and Makeni) reduced our sample size to 1973 participants from a target of 2100. Our statistical power calculations assumed an 18-month attrition rate of 20% based on historical data, which would yield a final cohort of 1578 participants (132 per clinic), as opposed to 1680 (140 per clinic), after 18 months of follow-up. This reduction in the size of the analysis cohort, if present, will have a minimal effect on our statistical power due to the cluster randomized design, whereby the number of clusters and the coefficient of variation between clinics exerts a greater impact on statistical calculations than the number of participants per clinic. Assuming 132 participants per clinic remain at 18 months and the estimated coefficient of variation of 0.14 does not change, we would still be able to detect a hazard ratio of 0.637 or lower, as opposed to 0.642 or lower, as a consequence of utilizing routine VL monitoring in clinical care.

Losses to follow-up are an important source of bias in prospective studies, and lost participants may differ from those continuing follow-up. We estimated an 18-month attrition rate of 20% from historical data, but estimates of mortality and attrition rates should account for potential changing norms among future patient populations and the effect of study participation. We attempted to minimize bias from ‘contamination’ effects – for example, if participants from control clinics switched to intervention clinics to take advantage of additional clinical services. Given that control arm patients are aware of, but do not receive, additional clinical services, there may be increased loss in this arm.

The investigators recently decided to extend the follow-up period of participants in the VLS to 36 months to accrue additional data on mortality, virologic failure, and other outcomes in this unique cohort. This extension will better inform a range of secondary research questions, including differences in immunologic response and clinical disease progression between study arms; the impact of more frequent ART regimen switching in the setting of limited available third-line and beyond treatment regimens; the incidence and pattern of accumulated HIV resistance mutations; and the cost-effectiveness of the two management strategies. Participant retention will be critical to maximizing study yield over this longer period, while a divergence in study clinic mortality rates, with a corresponding increase in the coefficient of variation, could adversely affect statistical power. The investigators intend to disseminate the study findings at the completion of the 36 month trial, rather than reporting interim 18 month findings, to avoid a potential effect on clinician behavior or the validity of the final results.

Overall, VLS has responded well to unforeseen circumstances, but additional resources will need to be committed to extend participant follow-up. Close collaboration and frequent reporting between investigators, senior research staff, and clinic-based staff is a necessity to ensure scheduled data and specimen collection, especially given the constraints of local clinical, laboratory, and transport infrastructure, and the difficulties of locating participants in the community. The VLS cohort will provide a range of important data on the monitoring and long-term outcomes of patients on ART in resource constrained settings.

## Supporting Information

Protocol S1 Viral Load Study trial protocol
(0.24 MB DOC)

Checklist S1 CONSORT checklist
(0.19 MB DOC)
