# Correction: A Cluster Randomized Trial of Routine HIV-1 Viral Load Monitoring in Zambia: Study Design, Implementation, and Baseline Cohort Characteristics

**DOI:** 10.1371/annotation/dbc3ee9b-dab4-494b-87d1-7c32b8d684b5

**Published:** 2010-03-23

**Authors:** John R. Koethe, Andrew O. Westfall, Dora K. Luhanga, Gina M. Clark, Jason D. Goldman, Priscilla L. Mulenga, Ronald A. Cantrell, Benjamin H. Chi, Isaac Zulu, Michael S. Saag, Jeffrey S. A. Stringer

The following statement was omitted from the Funding section of the article and should be added: The Viral Load Study is supported by the President's Emergency Program For AIDS Relief (PEPFAR) though a supplement to the National Institutes of Health (U01-AI069452; http://grants.nih.gov/grants ).

