# Supplementary material for: A Cluster Randomized Trial of Routine HIV-1 Viral Load Monitoring in Zambia: Study Design, Implementation, and Baseline Cohort Characteristics
Source: PLoS One. 2010 Mar 12;5(3):e9680. doi: 10.1371/journal.pone.0009680 (PMC2837376; doi:10.1371/journal.pone.0009680)
Supplement: Protocol S1 — Viral Load Study trial protocol (0.24 MB DOC) [file pone.0009680.s001.doc]

**Effectiveness of HIV Viral Load Monitoring**

**on Patient Outcome in Resource-Poor Settings**

**(Adult AIDS Clinical Trial Group -- CIDRZ Viral Load Testing Supplement)**

**17^th^ April, 2007**

**Version 2.0**

**Table of Contents**

[PRINCIPAL INVESTIGATORS/ CO-INVESTIGATORS 3](#__RefHeading___Toc120949572)

[STUDY TEAM MEMBERS 4](#__RefHeading___Toc120949588)

[ROLES of INVESTIGATORS 4](#__RefHeading___Toc120949589)

[Introduction 6](#__RefHeading___Toc120949591)

[Rationale/Background 6](#__RefHeading___Toc120949592)

[Summary of Ongoing and Planned Studies 6](#__RefHeading___Toc120949593)

[Study Justification 9](#__RefHeading___Toc120949594)

[Study Objectives 9](#__RefHeading___Toc120949595)

[Study Design 9](#__RefHeading___Toc120949596)

[Primary Hypothesis 10](#__RefHeading___Toc120949597)

[Design 10](#__RefHeading___Toc120949598)

[Patient Population 14](#__RefHeading___Toc120949599)

[Study Procedures/Methods 14](#__RefHeading___Toc120949600)

[Locations 14](#__RefHeading___Toc120949601)

[Study Inclusion Criteria 14](#__RefHeading___Toc120949602)

[Study Exclusion Criteria 14](#__RefHeading___Toc120949603)

[Participant Selection and Enrollment Procedures 15](#__RefHeading___Toc120949604)

[Costs to Participants, Reimbursements and Incentives 15](#__RefHeading___Toc120949605)

[Clinical Study Data Collection 15](#__RefHeading___Toc120949606)

[Monitoring for Therapeutic Failure 15](#__RefHeading___Toc120949607)

[Study Data Collection Points 17](#__RefHeading___Toc120949608)

Schedule of Evaluations……………………………………………………………………………………….18

[Lab methods and sample collection 19](#__RefHeading___Toc120949609)

[Viral load testing 19](#__RefHeading___Toc120949610)

[Storage of specimens 19](#__RefHeading___Toc120949611)

[HIV-1 resistance testing: 19](#__RefHeading___Toc120949612)

[Outcome Measures/Analysis 19](#__RefHeading___Toc120949613)

[Primary Measure of Outcome for Comparison between Groups 19](#__RefHeading___Toc120949614)

[Secondary Measure of Outcome for Comparison between Groups 20](#__RefHeading___Toc120949615)

[Statistical Considerations 20](#__RefHeading___Toc120949616)

[Sample size calculation 20](#__RefHeading___Toc120949617)

[Human subjects requirements 21](#__RefHeading___Toc120949619)

[Informed Consent 21](#__RefHeading___Toc120949620)

[Subject Confidentiality 21](#__RefHeading___Toc120949621)

[Protocol Compliance 21](#__RefHeading___Toc120949622)

Subject Risk………………………………………………………………………………………………………22

[Administrative Procedures 22](#__RefHeading___Toc120949623)

[Study Coordination 22](#__RefHeading___Toc120949624)

Protocol registrtion……………………………………………………………………………………………22

[Quality Control and Data Management 22](#__RefHeading___Toc120949625)

[Data Monitoring Committee 22](#__RefHeading___Toc120949626)

Adverse Event Reporting………………………………………………………………………………………23

[Plan for Dissemination of Information 23](#__RefHeading___Toc120949627)

ETHICAL CONSIDERATIONS……………………………………………………………………………………..23

REFERENCES:………………………………………………………………………………………………………25

## PRINCIPAL INVESTIGATORS/ CO-INVESTIGATORS

| **Birmingham, Alabama** | | |
| --- | --- | --- |
| Michael S. Saag, MD; Principal Investigator  Professor of Medicine  Director, UAB Center for AIDS Research (CFAR)  University of Alabama at Birmingham (UAB)  908 South 20th Street, CCB #142  Birmingham, AL 35294-2050  Phone: (205) 934-7349  Fax: (205) 975-6120  E-mail: [msaag@uab.edu](mailto:msaag@uab.edu) |  | |
|  |  | |
| **Lusaka, Zambia** | | |
| Jeffrey S. A. Stringer, MD, Protocol Co-Chair  Associate Professor, UAB OB/GYN  Director, Center for Infectious Disease Research in Zambia (CIDRZ)  P.O. Box 34681, Lusaka, Zambia Phone: 260-1-293-661, 293-772 (office) Phone: 260-96-860-604 (mobile)  [stringer@cidrz.org](mailto:stringer@cidrz.org) | | Peter Mwaba, MD, PhD; Protocol Co-Chair  Consultant Physician  Academic Chair, Department of Medicine  University Teaching Hospital  Department of Medicine  Private Bag RWIX, Lusaka, Zambia  Phone: 260-97-775354 (mobile)  [pbmwaba@yahoo.com](mailto:pbmwaba@yahoo.com) |
| Isaac Zulu, MD, MPH  Consultant Physician  Clinical Chair, Department of Medicine  University Teaching Hospital  Department of Medicine  Private Bag RWIX, Lusaka, Zambia  Phone: 260-95-751-428  [zuluisaac@yahoo.com](mailto:zuluisaac@yahoo.com) | | Ron Cantrell, MS  Epidemiologist  UAB-CIDRZ  P.O. Box 34681, Lusaka, Zambia Phone: 260-1-293-783, ext 201  [cantrell@cidrz.org](mailto:cantrell@cidrz.org) |
| Jens Levy, MS  Epidemiologist  UAB-CIDRZ  P.O. Box 34681, Lusaka, Zambia Phone: 206-1-293-661, ext 207  [jens@cidrz.org](mailto:jens@cidrz.org) | | Benjamin Chi, MD  Instructor & Fellow, UAB OB/GYN  UAB-CIDRZ  P.O. Box 34681, Lusaka, Zambia Tel: 206-1-293-661, ext 207  Email: [bchi@cidrz.edu](mailto:bchi@cidrz.edu) |
| Elizabeth Stringer, M.D.  Assistant Professor, UAB OB/GYN  UAB-CIDRZ  P.O. Box 34681, Lusaka, Zambia  Phone: 260-1-293-661, x133  E-mail: [eli@uab.edu](mailto:eli@uab.edu) | |  |

## STUDY TEAM MEMBERS

*UAB-Center for Infectious Disease Research in Zambia (CIDRZ)*

Stewart Reid, MD Medical Director, CIDRZ

Alain DeGroot Data Manager, CIDRZ

Mary Morris, MSN QA/QC Coordinator, CIDRZ

Dara Potter, MBA, DrPH Project Advisor, CIDRZ

Jason Goldman, BSE VLS Study Coordinator, CIDRZ

Dora Luhanga VLS Clinical Coordinator, CIDRZ

Rhoidah Chitambi VLS Senior Research Nurse, CIDRZ

## ROLES of INVESTIGATORS

Principal Investigators will be responsible for the overall conduct of the study, human subjects’ protection, coordination of funding streams, study design, and study implementation. Those based in Zambia will also manage local staff and ensure that proper procedures are implemented to notify participants of any new and important findings from this evaluation.

Abbreviations

Organizations/programs

CIDRZ Center for Infectious Disease Research in Zambia

PEPFAR President’s Emergency Plan for AIDS Relief

WHO World Health Organization

Technical terms

AIDS Acquired Immune Deficiency Syndrome

ART antiretroviral therapy

ARV antiretroviral

HAART highly active antiretroviral therapy

HIV human immunodeficiency virus

TB tuberculosis

# Introduction

# Rationale/Background

Like many countries in sub-Saharan Africa, the scale-up of HIV care and treatment services in Zambia has accelerated tremendously over the past 18 months.^1^ Much of this activity has been concentrated in Lusaka, through collaborations between the Centre for Infectious Disease Research in Zambia (CIDRZ) and the Zambian Ministry of Health.^2^ Through November 2005, we have enrolled 30,000 HIV-infected patients across 18 sites, over 19,000 of whom have started antiretroviral therapy (ART). At our current rate, approximately 2,000 new participants enroll for HIV care each month and 1,000 start ART.

As more individuals start ART in sub-Saharan Africa, it is critical that we incorporate effective, proven algorithms for clinical monitoring; without them, the impressive decreases in morbidity and mortality owed to ART could be off-set by treatment failure and population-level ARV drug resistance. Despite its key role in HIV care in the developed world,^3^ the utility of routine viral load in this setting is a subject of debate; in particular, it remains uncertain whether the limited resources for care are best spent on viral load monitoring or on the purchase of therapy for more patients. Central to these discussions are the yet unknown long-term benefits and cost-effectiveness of HIV viral load monitoring in low-resource settings like Zambia. While the use of HIV viral load would undoubtedly increase the rate of ARV drug regimen utilization, it is possible that, in settings with high prevalence of opportunistic infections^4,5^ and limited repertoires of antiretroviral agents, the benefits may not be as striking or cost-effective when compared to the United States or Europe.

We propose to examine this critical research question. Using a community-randomized design, we will evaluate the effectiveness of routine HIV viral load monitoring on patient survival. This targeted evaluation will not only provide critical information on the potential survival benefits of viral load monitoring in a resource-poor setting, it will also provide urgently needed information on feasibility, acceptability, and cost-effectiveness as well.

## Summary of Ongoing and Planned Studies

CIDRZ was formed in 1999 as collaboration between the Zambian government and researchers from the University of Alabama at Birmingham (UAB). The organization currently has a robust research portfolio: over 1,500 active research participants are enrolled in a variety of clinical research studies, including 3 DAIDS-sponsored network trials. CIDRZ is also deeply engaged in a partnership with the Zambian government to provide both prevention of mother-to-child HIV transmission (PMTCT) and ART services across 4 of the country’s 9 provinces. Through November 2005, the 61 CIDRZ-supported sites had provided PMTCT services to over 150,000 women.^6,7^ In addition, we had enrolled more than 30,000 HIV-infected individuals into our long term care and treatment initiatives, 19,000 of whom were started on ART.^2^

HIV treatment in Zambia

UAB/CIDRZ was awarded funding through the U.S. Emergency Plan for AIDS Relief (PEPFAR) to support the Zambian government in its scale-up of HIV care and treatment in Zambia, as part of an Elizabeth Glaser Pediatric AIDS Foundation consortium. In our original application, we proposed the initiation of ART for approximately 12,000 Zambians over the 5-year award, in sites in Lusaka, Western, and Southern provinces.

Thus far, enrollment has far exceeded original expectations. Through a close relationship with the Ministry of Health, National AIDS Council, and experts at the University Teaching Hospital (UTH), we have assisted the Lusaka District HIV Care and Treatment Program since its April 2004 inception. This program was Zambia’s first effort to provide ART at the primary healthcare level; prior to it, care was only available at the UTH and other provincial hospitals. The Lusaka District program started in 4 health care facilities, but has rapidly expanded to 11 sites. We also have been able to provide drug and reagent support to the UTH. By early 2006, we will be supporting 15 clinical care sites in Lusaka.

We are currently supporting government program sites outside of the capital city of Lusaka as well. We began assisting HIV care and treatment at Lewanika General Hospital in Mongu (Western province) and Chikankata Mission Hospital (Southern province) in 2004 and will soon begin supporting Livingstone General Hospital (Southern province). In addition, we have been given additional PEPFAR funds through the local U.S. CDC mission to scale-up services in Eastern province hospitals in Chipata, Katate, and Petuake.

The CIDRZ PEPFAR team offers comprehensive support to all aspects of HIV care and treatment at all the sites we support, with the exception of the UTH, which needs less technical assistance. The CIDRZ pharmacy department assists in drug forecasting and procurement. The CIDRZ laboratory currently offers essential laboratory tests such as CD4+ cell counts free-of-charge. The CIDRZ medical team plays a large role in clinician training and oversight. In our sponsored activities alone, we have trained more then 300 nurses, clinical officers (local equivalent of a nurse practitioner), and physicians on basic HIV care. An additional 200 nurses have been trained in adherence counseling. CIDRZ monitoring team has used the PTS software to produce weekly and monthly reports to both the Zambian government and our donors. With support from the Zambian government, we have been able to offer all these services, including antiretroviral drugs, free-of-charge.

Data collection

The Lusaka medical record system has been key to the success of the program,^2^ and has been a model for other organizations developing ART services in Zambia. The system is founded on a comprehensive set of clinical care forms based on the Zambian national guidelines for HIV care. Specifically developed by CIDRZ, each form guides the clinician through a routine HIV-related visit and facilitates appropriate clinical management.

The Patient Tracking System (PTS) builds its database upon information gathered in these forms. Each patient visit generates a paper form; select data from that form are immediately entered by data entry staff at the end of the patient visit. Currently, all 11 HIV clinics have at least one computer that runs PTS; sites with several thousand active patients operate up to three. Data from the Lusaka sites are instantly available via a wireless wide-area network developed by CIDRZ; staff members from remote sites bring data on storage disks weekly. PTS allows us to track patient visits, monitor program growth, manage drug forecasting, and generate reports for funding agencies. A rudimentary version 1.0 of the PTS software was rapidly installed at the onset of clinical care delivery in April 2004 to meet immediate programmatic needs. Our team then refined the data model to create the current version 2.2. We now collect approximately 200 individual data elements – roughly two-thirds of the data entered on the clinical forms – in standard fashion, regardless of the program setting. Fields collected include: CD4+ cell count, WHO stage, pregnancy status, method of contraception, previous ARV use, current ART regimen, drug manufacturer, drug adherence, weight, body mass index, and incident co-morbidities such as tuberculosis infection. When HIV viral load becomes available in March 2006 at CIDRZ Central Laboratory, this data will be entered into PTS as well.

Medical oversight

Currently, medical oversight is provided by a team of 8 physicians and 8 nurses. Trained specifically in HIV care and treatment, these healthcare providers rotate through CIDRZ-supported HIV treatment sites. Each physician split his/her time between 2 assigned clinics, assisting the regular staff with difficult medical cases and training full-time clinical officers. Nurses are responsible for monitoring of clinical care through detailed chart review. Currently, 10% of all clinical charts are reviewed for completeness and adherence to the Zambian national treatment guidelines. Each month, reports are generated and clinical staff is given feedback regarding commonly noted problems. In addition, weekly team meetings are held by local CIDRZ experts and visiting clinicians from UAB and the University of California at San Francisco. The purpose of this meeting is to review complex cases at each facility and to review training topics in long-term HIV care and treatment.

Although we have developed an adequate medical oversight infrastructure for programmatic purposes, we recognize the higher standards of data completeness and patient ascertainment needed in epidemiologic research. In October 2005, UAB-CIDRZ was a recipient of the Doris Duke Charitable Foundation’s ORACTA grant (B.Chi, PI). This 2-year, $200,000-dollar award will be used to bolster the PTS database in various ways, including: (1) enhancing the current PTS software to include data logic checks and decision support, (2) increasing staff for the monitoring of clinical care, (3) improving upon the existing system for ascertainment of patient outcomes following missed visits, and (4) improving the ability to determine cause of death, by incorporating verbal autopsy interviews into follow-up visits. These activities are meant to augment the QA/QC and monitoring aspects of this protocol and further ensure high-quality data for program analysis.

Other studies

Because of the comprehensive nature of this community randomized trial, it is important to note other on-going clinical trials, particularly ones recruiting from similar target populations. Currently, CIDRZ is conducting two such trials in Lusaka district clinics.

- **ACTG 5208** is an NIAID/DAIDS-sponsored randomized controlled trial comparing treatment response to NNRTI- or protease-inhibitor-based ART regimens in women with and without previous exposure to single-dose nevirapine (NVP) for PMTCT. A total of 60-80 women will be enrolled from Lusaka from a single site: Kalingalinga Clinic.
- **CDC-RX** is a multi-center clinical trial investigating the effect of previous NVP exposure for PMTCT on treatment response to later NNRTI-containing ART regimens. This observational trial is based in two clinics (Kanyama and Matero Reference) and will enroll 400 women. Completion of the enrollment phase is anticipated in mid 2006.

Because of the specificity of the target populations and because of their relatively small samples in comparison to the overall program size, we do not believe these trials will significantly affect either recruitment or analysis for this study.

# Study Justification

The rapid roll-out of ART in resource limited settings has been accomplished with remarkable success. Owing to the overwhelming need to treat as many individuals as possible and the relative cost and lack of availability of viral load testing in most resource limited countries, the early launch of PEPFAR programs focused on delivery of drug therapy without the use of viral load testing. A potential consequence of not using viral load testing routinely in this setting is a higher risk of resistant virus, owing to ongoing replication in the setting of selective drug pressure. Should resistance develop there is a higher likelihood of regimen failure and ultimately clinical progression. In addition, there may be increased de novo transmission of drug resistant virus, leading to public health concerns. On the other hand, even in resource rich countries like the United States, there has never been a study demonstrating a survival advantage to the use of viral load testing in clinical practice. Moreover, tension exists regarding the use of scarce resources on testing modalities versus the purchase of drugs to treat more patients. Taken together, there is equipoise with respect to the use of routine real-time viral load testing in resource constrained settings. Therefore, an evaluation is desperately needed to inform clinicians and policy makers regarding the use of viral load testing in these settings.

Study Objectives

**Primary Objective:**

1. To assess mortality at 18 months among ARV naïve patients who will be initiating ARV therapy and receiving care at facilities with access to routine HIV viral load testing compared to those initiating first regimens and receiving care at facilities with discretionary viral load testing.

**Secondary Objective:**

1. To compare indicators of clinical disease progression in the two comparison groups, including:
   1. Incident opportunistic infections
   2. CD4+ response
   3. Weight
2. To assess the incidence and degree of drug resistance development in each group.

**Tertiary Objectives:**

1. To assess the feasibility, acceptability, and cost effectiveness of the two management strategies in a resource-poor sub-Saharan African setting.

# **Study Design**

Using a community-randomized design, we will assign clinics offering HIV care and treatment in the Lusaka health district to one of two arms for the monitoring of antiretroviral therapy:

1. **Clinical Monitoring with discretionary viral load testing:** utilizes the current Zambian standard of care (CD4+ lymphocyte response and clinical status) to determine treatment failure, with viral load testing used sparingly to adjudicate uncertain clinical presentations;
2. **Routine Viral Load Monitoring:** incorporates routine, periodic HIV viral load testing into clinical care and decision-making, in addition to the current standard of care

Utilizing a comprehensive data collection and patient tracking system created by our group in Zambia (see Appendix), we will compare mortality among patients managed with routine viral load monitoring versus those managed without routine monitoring. This primary study outcome will be measured at 18 months of follow-up. In addition, we will measure incident opportunistic infections and other markers of clinical disease progression among patients in each arm.

## Primary Hypothesis

Our primary hypothesis is that a strategy of routine viral load monitoring will result in improved patient survival (33% reduction in mortality) at 18 months post treatment when compared to the current monitoring strategy of discretionary viral load testing reflexive to clinical and CD4+ lymphocyte counting criteria.

**Secondary Hypothesis**

1. Our secondary hypothesis is that a strategy of routine viral load testing will result in less antiretroviral drug resistance at 18 months post therapy initiation as well as evidence of reduced clinical disease progression, as determined by
   1. incidence of new opportunistic infections
   2. CD4+ lymphocyte response at 6, 12, and 18 months post therapy initiation
   3. Patient weight response at 6, 12, and 18 months post therapy initiation

**Tertiary Hypotheses**

Our tertiary hypotheses are that:

1. routine viral load testing will be feasible in our low-resource setting where the clinical staff comprises mostly non-MD physicians “extenders” (clinical officers and nurses); and
2. routine viral load testing is cost-effective in resource limited settings

## Design

We propose a two-arm, community-randomized targeted evaluation in which clinics are assigned to the standard of care for monitoring HIV treatment failure in Zambia (evidence of clinical progression, CD4+ cell count, and the new intervention of discretionary viral load monitoring) or a modified strategy that incorporates routine HIV viral load testing.

Current clinical care for HIV in Lusaka district sites

Patients are referred to the HIV care and treatment program with documented HIV seropositivity. Initial evaluation consists of a demographic profile, medical history, and physical examination. Initial laboratory evaluation includes CD4+ lymphocyte counting (Beckman Coulter Epics XL-MCL 4-color Flow Cytometer) and complete blood count (Sysmex XT2000i Analyzer). If patient report or physical exam suggests renal or hepatic disease, plasma is drawn for serum creatinine or liver transaminases, respectively (Roche COBAS Integra 400+).

A follow-up appointment is made for 1-2 weeks later, at which time lab results are reviewed and ARV eligibility determined. Eligibility criteria for ART initiation are based on Zambian National Guidelines for HIV treatment. In the first months of the program, ART was initiated on individuals with either CD4+ cell count < 200 cells/mm^3^, or WHO stage III or IV. In 2005, these guidelines were revised to exclude ART eligibility for patients in WHO Stage III with CD4+ cell count > 350 cells/mm^3^. Those not meeting ART criteria are classified as either intermediate HIV disease (requiring routine medical visits every 3 months) or early HIV disease (requiring routine medical visits every 6 months). At these follow-up appointments, a re-evaluation by physical exam and CD4+ cell count (every 6 months only) is performed to determine progression of disease and need for ART.

For individuals meeting the criteria for ART, baseline laboratory tests are drawn – complete blood count, liver transaminases, and creatinine – and adherence counseling is performed. An assessment is then made regarding co-trimoxazole prophylaxis. For those with CD4+ cell count < 200 cells/mm^3^ or in WHO Stage IV, co-trimoxazole is administered for 2 weeks prior to initiating ART; this strategy of staggering drug initiation allows the clinician to differentiate between common adverse effects (e.g. rash) of co-trimoxazole from that of antiretroviral drugs, such as nevirapine (NVP). ART is started after these 2 weeks, with a strict follow-up schedule of 6 visits over the first 3 months, focusing on adherence and possible adverse events. Routine laboratory surveillance includes hemoglobin and liver transaminase evaluations at 3 months for all patients; in urgent or emergent cases, tests are performed as deemed necessary by the clinician. For individuals on a zidovudine (ZDV)-based regimen, hemoglobin levels are monitored more closely, with scheduled checks at post-initiation weeks 2, 4, and 8 as well. Routine clinical follow-up of these patients occurs every 3 months, with CD4+ lymphocyte count performed every 6 months. All patients starting ART are offered enrollment into a district-wide program for home-based, adherence monitoring, similar to local models for directly observed tuberculosis therapy.

First line drug regimens are lamivudine (3TC) plus NVP plus either ZDV or stavudine (D4T). We have commingled drugs procured by the Ministry of Health (generic or “copy” ARVs[8]) with drugs procured by the U.S. government (until recently,[9] brand name drugs only). Various double- and triple-fixed-dose combinations are available. Brand and copy formulations are used interchangeably, sometimes for the same patient, but efforts are made to continue patients on the same regimens from month-to-month where possible; however, the regimens remain the same regardless of whether brand name or generic drugs are used. We start ZDV or D4T-based regimens based mostly upon drug availability, but avoid ZDV in anemic patients (hemoglobin < 10). Individuals placed on a NVP-based regimen are started on a half dose over the first two weeks, followed by escalation to the full dose after week 2. To avoid interactions between NVP and rifampin, we defer ART for patients receiving acute phase therapy for tuberculosis, unless their CD4+ cell count is < 50 cells/mm^3^, in which case we start ART immediately with an efavirenz (EFV)-based regimen.

Patients doing well on therapy are seen every 6 months by a clinician for history and physical exam, weight monitoring, and repeat CD4+ cell count. A follow-up appointment is made within 1-2 weeks to review the CD4+ result with the patient. Patients on ART present monthly to the clinic to collect their ARVs. Pharmacy staff administers symptoms questionnaires and refer patients to the clinical side who may need clinical evaluation. Walk-ins and “sick calls” are also taken each day.

Determination of treatment failure is based on WHO staging and CD4+ cell count. Individuals who are judged to be adherent to their prescribed regimen and who meet any of the following criteria are switched to a second-line drug regimen according to criteria for Monitoring for Therapeutic Failure (see section below).

Because of local resource constraints, neither HIV viral load testing nor ARV resistance testing has been incorporated into this clinical monitoring prior to the study initiation. Second-line therapy is tailored for each patient, though commonly used agents include protease inhibitor based therapy (lopinavir/ritonavir, indinavir/ritonavir, atazanavir/ritonavir, or nelfinavir, with concomitant changes in the nucleoside backbone.

Allocation of clinical sites

This targeted evaluation will take place in 10 sites across the Lusaka district, all with pre-existing, PEPFAR-sponsored programs for HIV care and treatment. Patient flow, staffing, and clinical protocols have been standardized across all sites as part of the ongoing program. Administrative support and medical oversight are provided centrally by CIDRZ.

Patient flow and activities

Five clinics will be selected for evaluation of feasibility and effectiveness of routine viral load monitoring; five will be selected as controls. In all clinics, care will proceed according to the standards set forth by the Zambian national guidelines for HIV care. Clinical monitoring will include WHO staging and CD4+ cell count trends, as described above. Targeted evaluation activities will be minimally intrusive to current patient flow and management. No change will be made to visit schedules.

The first 175 new-start patients at each intervention clinic who provide written informed consent to participate in the targeted evaluation will be managed according to a specified protocol that includes HIV viral load at study enrolment, followed by viral load testing at 3 months and with each CD4+ determination (6 months, 12 months, 18 months, and when indicated by the clinician). This group of patients will be referred to as the “evaluation cohort.”

Patients receiving care at the evaluation clinics (but not part of the evaluation cohort) will have access to targeted use of viral load testing. HIV viral load will be available for use at the clinician’s discretion, when treatment failure is suspected according to clinical and immunologic (i.e. CD4+ cell count) criteria.

In the evaluation clinics, routine and discretionary viral load tests will be returned within an estimated 1 week of collection. When drawn at the same time as CD4+ cell count (or other tests), patients will be given an appointment to return in 1-2 weeks, according to the regular clinical protocol. If a blood specimen is drawn separately, as in the case of suspected treatment failure, an appointment is made after a similar interval. In the case of missed visits, community health workers will be deployed to trace late patients according established procedures.

Outreach Workers

Each site has a group of paid staff and volunteers who go out into the community and bring patients into clinic who have transportation problems or who have missed clinic visits. These outreach efforts reduce the lost to follow up rates dramatically and will be used routinely during the course of the study at every participating clinic.

Data collection

The Lusaka medical record system^10^ has been key to the success of our program,^2^ and has been a model for other organizations developing ART services in Zambia. The system is founded on a comprehensive set of clinical care forms based on the Zambian national guidelines for HIV care (see Appendix). Specifically developed by our group, each form guides the clinician through a routine HIV-related visit and facilitates appropriate clinical management. In this fashion, each patient visit generates a paper form; select data from that form are immediately entered into a computerized database by data entry staff at the end of the patient visit. We now collect approximately 200 individual data elements – roughly two-thirds of the data entered on the clinical forms – in standard fashion, regardless of the program setting. Fields collected include: CD4+ cell count, WHO stage, pregnancy status, method of contraception, previous ARV use, current ART regimen, drug manufacturer, drug adherence, weight, body mass index, and incident opportunistic infections.

Currently, all 11 HIV clinics have at least one computer that runs our custom-designed Patient Tracking System (PTS) software; sites with several thousand active patients operate up to three. Data from the Lusaka sites are instantly available via a wireless wide-area network developed by CIDRZ. PTS allows us to track patient visits, monitor program growth, manage drug forecasting, and generate reports for funding agencies.

Because of the comprehensive nature of its data gathering and its deployment to all Lusaka clinic sites, we will rely on PTS for data collection in this targeted evaluation. A critical feature of PTS is the ability to generate datasets specific to time periods and filter parameters (e.g., clinic and informed consent) and devoid of personal identifiers such as name. Because PTS is currently a service (versus research) database, however, we have added provisions in this proposal to ensure data quality and completeness. This includes the hiring of 5 data associates to double-enter 20% of the clinical charts at our 10 sites in a separate QA database. A data supervisor will also be employed to compare the two databases and generate reports regarding data integrity. Discrepancies will be followed up by our data associates and resolved in the main PTS database. These activities should significantly bolster data quality; however, they should not disrupt clinical care.

Monitoring the quality of clinical care

In addition, we will deploy a roving team of 5 well-trained QA/QC nurses to assure quality of care at each site, through weekly chart audits and follow-up of inconsistent data generated by PTS reports. In our 3 NIAID-supported clinical trials, we have trained such nurses, now experienced with network-caliber trials, who are available for this project. A pre-specified percentage of patient visit forms will be reviewed from each site (e.g., between 15-20% of weekly visits), although these proportions may change based on the results of the ongoing reviews. Our QA/QC nurses will be integrated into the clinical oversight team (currently comprising 6 doctors and 4 nurses), substantially bolstering the existing QA/QC system and improving clinical care.

**Patient Population**

Male and female HIV-infected adults initiating ART in UAB-CIDRZ supported health centers in Lusaka who meet inclusion criteria will be eligible for study participation.

# Study Procedures/Methods

## Locations

The study will be conducted in 10 public sector clinics in Lusaka, Zambia. Each facility operates under the auspices of the Lusaka District Health Management Team and is providing HIV care and treatment services with support from UAB-CIDRZ. Immediately prior to facility randomization, we will assess mortality rates at each facility to determine whether matching by this indicator will be necessary.

## Study Inclusion Criteria

1. Documented HIV-1 infection (according to local standard rapid testing algorithms);

1. Adults 18 years of age or greater who are able and willing to provide informed consent to participate;
2. Qualifying for antiretroviral therapy per Zambian national guidelines, which are any of the following:
   1. CD4+ lymphocyte count less than 200 cells /mm^3^; or
   2. WHO Stage IV disease; or
   3. WHO Stage III disease **and** CD4+ lymphocyte count less than 350 cells / mm^3^;
3. Residence in the geographical catchment area of the VLS clinic and intent to remain in current geographical area of residence for the duration of study;
4. Willingness to adhere to the study visit schedule, and to be followed-up at home in the event of a missed study visit;
5. Initiating ART on the day of VLS enrollment, informed consent and baseline blood draw.

## Study Exclusion Criteria

1. Receipt of more than 7 days (cumulative) of prior antiretroviral therapy at any time prior to study entry, with the exception of ZDV prophylaxis or single dose NVP for prevention of mother-to-child transmission (PMTCT).
2. Any exposure to antiretroviral therapy in the past one month will be grounds for exclusion from the study.
3. Any condition that in the opinion of the investigators would interfere with adherence to study requirements. Such conditions include mental illness or active drug or alcohol use or dependence.
4. Serious illness requiring referral to UTH at the time of treatment initiation.
5. For patients seeking care at sites randomized to clinical / CD4 monitoring only: participation in another research protocol that offers routine viral load testing.
6. Does not consent to all aspects of study protocol including serum storage.

## Participant Selection and Enrollment Procedures

Prior to study implementation, the protocol and consent forms will be approved by DAIDS, by the IRB at the University of Alabama-Birmingham, and by the University of Zambia Research Ethics Committee. Once a candidate for study entry has been identified, details will be carefully discussed with the participant. The participant will be asked to read and sign the consent form. If the participant and legal guardian are unable to read, the process for consenting illiterate participants, as defined by the University of Zambia Research Ethics Committee, will be followed.

## Costs to Participants, Reimbursements and Incentives

Study participants will not be reimbursed for regularly scheduled study visits. All laboratory testing for the study, including viral load and CD4+ lymphocyte counts will provided free of charge.

## Clinical **Study Data Collection**

Clinical variables to be collected at initial and follow-up visits include:

- Demographics, height/weight
- Presence of clinical symptoms of AIDS based on WHO criteria (clinical HIV stage)
- Presence of TB and confirmation by smear or culture
- Dispensing, receipt, tolerance, and adherence of ARV therapy
- Receipt of treatment for other opportunistic infections
- Death and cause of death

## Monitoring for Therapeutic Failure

Partial ARV regimen changes may occur for reasons of toxicity, intolerability, or drug interactions at the discretion of the clinician. Patients deemed to have failed therapy will be changed to a second line regimen according to the Zambian National Guidelines. Criteria for failure will differ by intervention arm as follows:

Criteria for therapeutic failure (clinical monitoring with discretionary viral load arm):

1. New or recurrent WHO stage 3 or 4 condition while on ART for > 6 months. Discretion by physician will be needed to determine which WHO stage 3 or 4 events constitute disease progression;
2. Less than 50 cells / mm^3^ increase in CD4+ lymphocyte count at 6 months of therapy in the absence of marked clinical improvement;
3. Absolute CD4+ lymphocyte count less than 100 cells / mm^3^ at 12 months of therapy;
4. More than 30% decline in CD4+ lymphocyte count from its peak post-treatment initiation value;
5. Decline in CD4+ lymphocyte count to value below that of treatment initiation;

- When Clinical or Immunologic criteria for therapeutic failure are met, a procedure for allocating “Discretionary” viral load testing will be utilized: Active infections will be appropriately investigated and treated. The CD4 count will be repeated one month after treatment of active infection or flagging for suspected therapeutic failure based on immunologic criteria. If the patient still meets criteria for therapeutic failure based on clinical or immunologic definitions after adherence is judged to be excellent and the CD4 count in repeated, then Viral Load testing will be pursued for those patients meeting either clinical or immunologic criteria, but not both. Patients meeting both clinical and immunologic criteria for therapeutic failure will be assumed to be in virologic failure and the decision to change to 2^nd^ line therapy will be made without Viral Load Testing. In those patients receiving Discretionary Viral Load testing, decisions to switch ART will be made based on criteria for Virologic Failure (below).
- NB: Immune Reconstitution Syndrome (IRIS), especially secondary to tuberculosis (e.g., lymphadenitis, will NOT be considered evidence of clinical progression and will be managed according to Zambian National Guidelines. IRIS is defined as an inflammatory process, characterized by clinical symptoms of fever, malaise, constitutional symptoms, and /or signs of local inflammation at the site(s) of involvement that respond to symptomatic treatment and does not require specific therapy directed toward the suspected underlying pathogen. The assessment of whether a clinical event represents IRIS or a genuine incident Opportunistic Infection will be determined locally by the attending physician in the clinic and adjudicated in a blinded fashion by the Data Management Committee (see below).
- NB: Certain WHO clinical stage 3 conditions (e.g. pulmonary TB, severe bacterial infections), may be an indication of treatment failure and thus require consideration of second-line therapy. Some WHO clinical stage 4 conditions (lymph node TB, uncomplicated TB pleural disease, oesophageal candidiasis, recurrent bacterial pneumonia) may not be indicators of treatment failure and thus do not require consideration of second-line therapy.

Criteria for therapeutic failure (routine virologic monitoring arm):

1. Suboptimal reduction in plasma viremia after initiation of therapy defined as inability to suppress to:
   1. at least 1.0 log below baseline by week 12 of therapy;
   2. < 400 copies/mL by week 24 of therapy;
2. Re-appearance of plasma viremia (defined as > 400 copies / mm^3^) after suppression to undetectable, or
3. Among patients who have experienced virologic failure in more than 2 regimens and it is deemed that undetectable viremia is not possible: a confirmed return of HIV RNA to < 0.5 log (3-fold) of pre-therapy baseline value.

- NB: Patients meeting definitions for virologic failure will have a repeat viral load test at the discretion of the treating physician after intensive adherence counseling.

## Study Data Collection Points

Routine care procedures will be implemented by each site. Study data will be collected via existing clinical care forms already in use in each of the proposed facilities (see Appendix). There will be no additional study visits or blood draws, however additional blood may be collected at routine visits for archiving of plasma and cells. The study staff will work with the clinical staff to collect study specific data and specimens at the following time points.

Table: Schedule for Study Data Collection Time Points

| Schedule of Evaluations |
| --- |

| **Event** | **Screening**  **-60 days** | **Entry**  **wk 0**  **+/- 3 days** | **2 wks** | **4 wks** | **8 wks**  **+/-14 days** | **12 wks**  **+/- 30 days** | **6 Monthly Visits +/-**  **30 days** | **Premature Discontinuation** |
| --- | --- | --- | --- | --- | --- | --- | --- | --- |
| Demographics and SES factors assessment | X |  |  |  |  |  |  |  |
| History and physical exam^1^ | X | X | X | X | X | X | X | X |
| Disease stage classification | X | X | X | X | X | X | X | X |
| Adherence evaluation | X | X | X | X | X | X | X | X |
| Hematology^2^ | X |  | X (HB only) | X (HB only) | X (HB only) | X (HB only) | X (HB only) | X |
| Chemistries^3^ | X |  |  |  |  |  |  |  |
| Liver function testing^4^ | X |  |  |  |  | X (if indicated) | X (if indicated) | X |
| Urine pregnancy test^5^ | X |  |  |  |  |  |  |  |
| Syphilis testing (RPR) | X |  |  |  |  |  |  |  |
| Routine viral load^6^ |  | X |  |  |  | X | X |  |
| CD4+ cell counts^7^ | X |  |  |  |  |  | X | X |
| Plasma archiving | X |  |  |  |  | X | X | X |
| Cells archiving | X |  |  |  |  |  | X | X |
| Resistance Testing |  |  |  |  |  |  | X^8^ | X^8^ |

Key:

^1^History: clinical data from medical visit including: review of systems, concomitant medications, study medications, adherence for those taking study medications, weight, height, record signs and symptoms of HIV related pathology, diagnosis (HIV related, STD, other), potential adverse events

^2^Hematology: CBC, differential, platelets; hemoglobin only at week 2, 4, 8, 12, 3 monthly if on AZT

^3^Chemistries: electrolytes, CPK, BUN, creatinine, LDH

^4^Liver function tests: SGOT (AST), SGPT (ALT), Alk Phos

^5^Not required for women who had a tubal ligation post pregnancy; prn after screening as indicated

^6^ In patients receiving care in the routine VL clinics only.

^7^A second HIV RNA PCR and CD4 cell count will be drawn within 30 days to confirm failure prior to discontinuing or switching HAART regimens;

^8^ A resistance test will be performed at end of study (month 18 or at premature discontinuation) on all patients whose end of study viral load values are > 1000 c/ml, provided the specimen was obtained at the time the patient was still on ARV therapy.

## Lab methods and sample collection

Blood samples will be collected by study staff on site and transported to the CIDRZ Central Laboratory. Procedures for specimen collection, transport, processing, storage and shipping will be included in the laboratory procedures manual.

### Viral load testing

In all study facilities, irrespective of randomization arm, clinicians will have access to discretionary use of viral load testing, per the local standard of care. Patients who are suspected to be failing therapy, and who meet one of but not both of pre-defined clinical or CD4 criteria for therapeutic failure will be offered viral load testing. In clinics randomized to routine viral load use, viral load testing will occur at baseline (study enrollment = ARV initiation) visit, and at months 3, 6, 12, and 18. The viral load tests will be performed at the CIDRZ Central laboratory that is certified by VQA according to DAIDS protocols.

Viral load results will be used by the program for patient management in accordance with the local standards for care. Plasma specimens will be stored at the other times indicated to allow future testing of early viral load response.

### Storage of specimens

Two or three additional tubes of blood beyond that being drawn through the government ARV program will be obtained from subjects at each blood draw as indicated in Table 1 for later testing and will be stored (frozen) in-country. Plasma will be stored at these 5 blood draws and cellular pellets will be stored once. CIDRZ does not plan to export any blood product samples. At the end of the study period, if capacity in Zambia is still not available for needed testing, CIDRZ will re-approach the Zambian Research Ethics Committee on the issue of exporting samples.

### HIV-1 resistance testing:

HIV resistance testing will be performed utilizing stored plasma samples at a research laboratory that is DAIDS and CLIA certified to perform these assays. DAIDS protocols for resistance testing will be followed. If a facility that meets these criteria is available within Zambia at the time the analytic service is needed, the assays will be performed locally. Otherwise, we will re-approach the Zambian Research Ethics Committee regarding the shipping of samples out of Zambia for resistance testing.

## Outcome Measures/Analysis

The primary outcome of this study is time to death. Analysis will need to account for withdrawals and losses to follow-up, along with any community effects. Hazard rate ratios for the use of viral load in the clinical management of patients on ART will be estimated by Cox regression, stratified by matched clinic pairs. Data will be assessed through intention to treat analysis. A secondary analysis will be performed to account for any patients who cross-over to a clinic in the opposite study arm.

### Primary Measure of Outcome for Comparison between Groups

Mortality at 18 months is the primary outcome metric between groups for this study.

### Secondary Measure of Outcome for Comparison between Groups

Secondary analyses will compare clinical progression, clinical progression plus mortality as a composite outcome, and development of resistance development between groups.

# Statistical Considerations

## Sample size calculation

Based on data from six viral load study sites (Bauleni, Chawama, Chilenje, Chipata, George, and Mtendere) as of February 1, 2007, we have a relatively precise estimate of 18-month mortality of 15.6 per 100 person-years. However, mortality rates have varied somewhat from clinic to clinic ranging from 13.1 per 100 person-years in George to 20.4 per 100 person-years in Chipata. Thus, a community-randomized evaluation, as proposed here, would have to account for the lack of independence of subjects within clinic populations. Higher coefficients of variation correspond to greater similarity within clinics and thus require increased numbers of communities. The coefficient of variation, K, for mortality rates in these 6 clinics is 0.14. Matching clinics based on mortality rate and other clinic characteristics allows us to use a matched K, which will be lower than lower the unmatched K. In the figure below, we illustrate the detectable hazard ratio (alpha of 0.05 and beta of 0.20) with 6 clinics per arm, 175 patients per clinic, and a conservative estimate of the matched coefficient of variation.


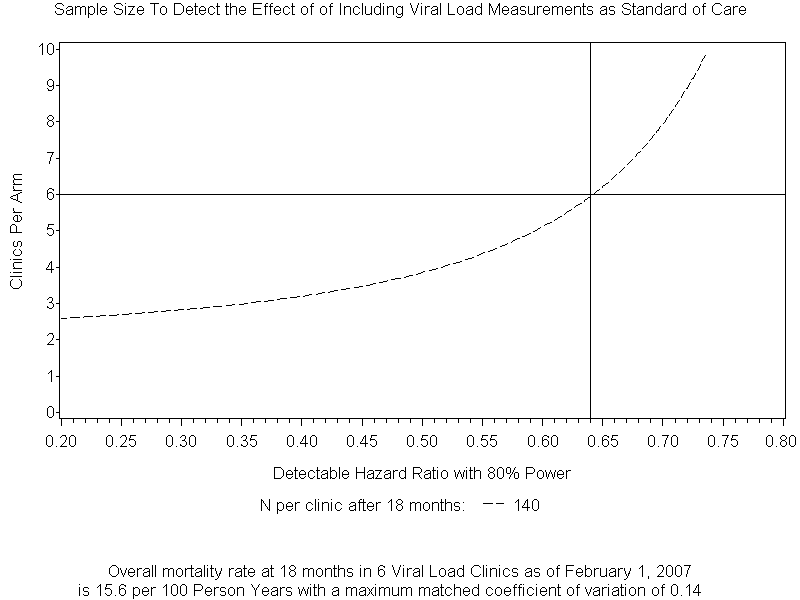


As demonstrated in the figure above, the number of clinics and the number of subjects within each clinic provides a minimum detectable hazard ratio of 0.64.  As such, we have chosen to conduct a community-randomized evaluation with 6 clinics per study arm, with 175 subjects per clinic. Assuming an attrition rate of 20%, we estimate that we will have 140 patients remaining in the study at each clinic at the end of 18 months of follow-up.  Using our observed unmatched K (0.14) as a conservative estimate of the matched K would give us the ability to detect a 36% reduction in mortality the mortality rate (15.6 per 100 person-years versus 10.0 per 100 person years) as a consequence of utilizing routine viral loads as part of clinical care. Our analysis will use proportional hazards modeling controlled by clinical sites.  Comparability between clinics will be assessed and utilized in the analysis.

# Human subjects requirements

The sites that participate in this study meet the requirements for the conduct of research using funds from the US Government. Each site has an Office for Human Research Protections (OHRP) approved IRB in country and a Federal Wide Assurance (FWA).

## Informed Consent

This protocol and the informed consent document and any subsequent modifications will be reviewed and approved by the Institutional Review Boards or Ethics Committees responsible for oversight of the study. Final approval and any modifications arising from these review processes will be reported to each review committee or board. The level of risk associated with this research is expected to be minimal. Written informed consent will be obtained from all study participants. The consent will be translated into the local language and back-translated back into English to assure accurate translation prior to study initiation.

## Subject Confidentiality

The confidentiality of all study records will be safeguarded to the extent legally possible. All laboratory specimens, reports, study data collection, process, and administrative forms will be identified by a coded number only, to maintain participant confidentiality. All local databases are secured with password-protected access systems, and computer entries will be identified by coded number only. Forms, lists, logbooks, appointment books, and any other listings that link participant ID numbers to other identifying information will be stored in a separate, locked file. Hard copies of data forms for data entry and analysis will be stored in a locked file when not in use. Specimens brought to the site laboratory or sent to other collaborating laboratories will be identified by unique laboratory specimen numbers and patient study numbers but not by patient names. Data management staff will be responsible for linking the results of the laboratory tests to the epidemiologic databases containing patient names and hospital numbers, and then transmitting that information back to the research staff and the collaborating physicians. All data analysis will be done on datasets which have only the study number as a unique identifier. Clinical information with individual identifiers will not be released without the written permission of the participant.

## Protocol Compliance

The study will be conducted in full compliance with the protocol. The protocol will not be amended without prior written approval by the protocol investigators and DAIDS. Protocol amendments requiring IRB approval must be submitted to the relevant ethic committees and approval obtained prior to implementing the amendment.

**Subject Risk**

Participation in this evaluation poses no additional risk for negative social impact than participation in the PEPFAR treatment program per se. There will be neither segregation of participants in this project nor any way to distinguish participants in this evaluation from those participating in the PEPFAR program. This concept is reinforced through the cluster randomization design whereby the entire clinic is assigned to the intervention rather than individuals.

# Administrative Procedures

## Study Coordination

The study will be coordinated by the Principal Investigators. The Principal Investigators will provide overall direction to the study and be responsible for oversight of co-investigators and study personnel at their sites. They will also be responsible for insuring that all necessary approvals and assurances are present and up-to-date at their sites. These tasks may be delegated to Co-Investigators and other study personnel.

**Protocol Registration**

Prior to a study site’s enrollment of its first patient, the site must formally register for participation in the study. This will include demonstration of the ability to conduct the study at the site, familiarity of the site personnel in study procedures and clinical trial conduct, and assurance of proper data collection and information management, including flow of information of viral load results and reporting. A checklist will be used to document the site’s readiness to participate in the protocol.

## Quality Control and Data Management

CIDRZ will develop or modify a data collection system to collect the necessary data elements for the study. Data entry and management will take place on-site. Aggregate data from all sites for the primary and secondary outcomes of the study will be coordinated by CIDRZ. Data will be reviewed periodically by study investigators for errors or variability in collection or entry.

## Data Monitoring Committee

A Data Monitoring Committee (DMC) will consist of a minimum of three members representing a range of expertise including biostatistics and HIV medicine. All members will be independent of the study investigators. The primary responsibility of the DMC will be to monitor study progress with respect to enrollment, follow-up, drop-outs and interim analyses.

If there is a difference between the arms with respect to response that is medically and statistically significant then the DMC will forward recommendations for possible study modification.

**Adverse Event Reporting**

This study utilizes existing, standard of care clinical facilities, procedures, and medication interventions that are part of the Zambian government Ministry of Health. Any adverse events due to medications or standard of care will not be considered Adverse Events related to involvement in this protocol. Rather, only those events that are a direct consequence of study participation, such as consequences of blood drawing or breeches of confidentiality, will be considered AEs in this study. Each clinic will be responsible for reporting Adverse Events to the study team. These events will be reviewed by both the study team and the Data Monitoring Committee at quarterly intervals or as needed.

# Plan for Dissemination of Information

The primary results of this study will be developed into a manuscript for publication in a peer-reviewed journal such as AIDS, Lancet, or other international journal with widespread readership that is appropriate for clinical infectious disease studies. The results may also be presented at international conferences on AIDS such as the Conference on Retroviruses and Opportunistic Infections, World AIDS Conference, International AIDS Society Conference, International Conference on AIDS and STDs in Africa, or other regional AIDS conference.

Data collected during the conduct of the trial may also be used to improve local services for providing HIV care and treatment in the host country. Abstracts about other facets of this program, such as operational issues related to study conduct, may be submitted for local, regional, or international conferences and published in peer-reviewed journals.

**ETHICAL CONSIDERATIONS**

We propose a community-randomized evaluation of routine HIV viral load testing in a setting where such testing is not normally available as part of routine (or even exceptional) care. Clinical care flow should remain unaffected; we will follow patients through standardized follow-up schedules, using an established data collection method that includes form-driven patient care protocols, for study information. In addition, we believe these activities fall under the auspices of our general programmatic consent form, which grants permission to use medical information (aggregated and stripped of personal identifiers) and left-over blood specimens for targeted evaluations. This consent form itself is currently under review at the local University of Zambia Research Ethics Committee (UNZA REC). We have utilized a similar consent process for MTCT-Plus in Lusaka and have this practice highly acceptable among patients.

We have chosen a community-randomized comparison because we do not feel it would be feasible to assign only a proportion of patients to use of viral load in a given clinic (but not others). In this spirit, we propose access to viral load testing in a “targeted” manner to all patients receiving care in “evaluation” facilities, as we study a more specific viral load utilization schedule within a smaller group. This will prevent the study from becoming prohibitively expensive while increasing accessibility to viral load testing at the clinician’s discretion.

Although use of HIV viral load has been shown to be beneficial in developed countries, its incorporation into resource-limited settings has not been recommended by international organizations such as the WHO. In such settings where salvage regimens are scarce and expensive, it is not known whether early detection of failure (rather than waiting for clinical evidence) will have a survival benefit. However, it is certain that such an intervention will have an associated incremental expense. Thus, without the critical information that we intend to gather through this evaluation, it will be impossible to make a meaningful estimate of the costs and benefits of such a strategy in settings of extreme resource constraint. We believe results from this targeted evaluation will greatly assist countries like Zambia in their policies and clinical protocols regarding HIV in the future.

**REFERENCES**
